# Direct Oral Anticoagulants or Warfarin for Left Ventricular Thrombus: A Meta-Analysis of Randomized Clinical Trials

**DOI:** 10.1016/j.jacadv.2025.102243

**Published:** 2025-10-21

**Authors:** David Koeckerling, Rohin K. Reddy, Joseph Barker, Volker Braun, James P. Howard, Yousif Ahmad, Lorenz Lehmann, Norbert Frey

**Affiliations:** a Department of Cardiology, Angiology and Respiratory Medicine, Heidelberg University Hospital, Heidelberg, Germany; b National Heart and Lung Institute, Imperial College London, London, UK; c Nuffield Department of Population Health, University of Oxford, Oxford, United Kingdom; d Medical Faculty Mannheim, University of Heidelberg, Mannheim, Germany; e Division of Cardiology, University of California, San Francisco, California, USA

**Keywords:** left ventricular thrombus, meta-analysis, direct oral anticoagulant, warfarin

**Figure:**
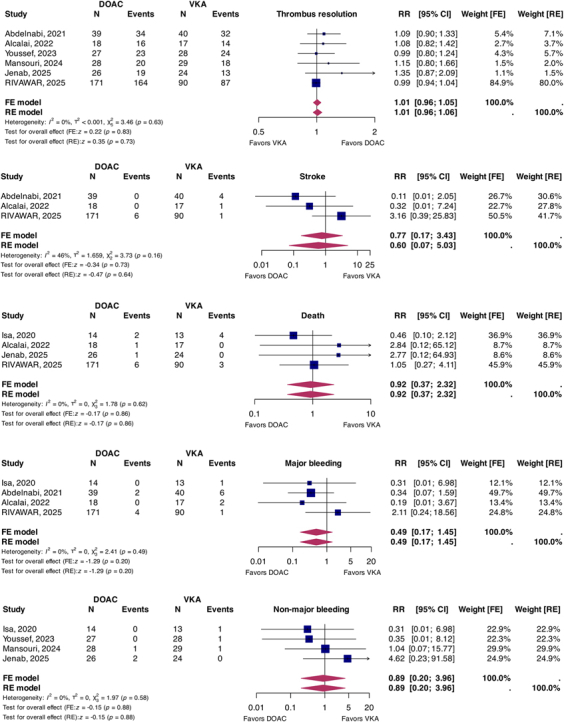

**What is the clinical question being addressed?**

What is the comparative efficacy and safety of vitamin K antagonists and direct oral anticoagulants in LV thrombus treatment?

**What is the main finding?**

The totality of available randomized evidence demonstrates no significant differences between VKAs and DOACs regarding thrombus resolution, all-cause mortality, stroke, major and nonmajor bleeding.

Left ventricular thrombi typically originate in regions of akinesia or dyskinesia due to severe ventricular dysfunction.^1^ Traditionally, vitamin K antagonists (VKAs) were considered the therapeutic standard to achieve thrombus resolution and prevent embolic complications. Direct oral anticoagulants (DOACs) have replaced VKAs for various indications due to their advantageous safety profile, simplicity of administration, and comparable efficacy. However, international guidelines reflect the scientific equipoise regarding anticoagulation regimes for left ventricular (LV) thrombi, as dedicated prospective trials in this setting are scarce.^2^ Previous meta-analyses reported advantages for DOACs regarding stroke, bleeding, and mortality, yet these studies primarily pooled data from nonrandomized observational studies, which are vulnerable to serious biases, particularly confounding.^3^ Recently, the Rivaroxaban vs Warfarin in Acute Left Ventricular Thrombus Following Myocardial Infarction trial reported outcomes in 261 patients with LV thrombus, effectively doubling the randomized evidence base.^4^ As such, we performed a prespecified meta-analysis of randomized controlled trials (RCTs) evaluating the comparative efficacy and safety of DOACs and VKAs in LV thrombus management.

This meta-analysis was performed according to Preferred Reporting Items for Systematic Reviews and Meta-Analyses guidance and registered on International prospective register of systematic reviews (CRD420251017074). CENTRAL, CINAHL, Embase, Medline, ClinicalTrials.gov, and World Health Organisation International Clinical Trials Registry Platform were systematically searched by a clinical librarian (V.B.) through March 2025 for RCTs comparing DOACs and VKAs in LV thrombus treatment and reporting prespecified endpoints. No language restrictions were applied, conference abstracts were not excluded. Abstract screening, data extraction, and risk-of-bias assessment were performed in duplicate (D.K., J.B.) with disputes resolved by consensus. Study-level quality assessment was conducted using Cochrane’s Risk of Bias 2 tool. Prespecified efficacy outcomes were LV thrombus resolution and stroke; prespecified safety outcomes were all-cause mortality, major bleeding, and nonmajor clinically relevant bleeding (International Society on Thrombosis and Haemostasis criteria). Since this is a study-level meta-analysis, no Institutional Review Board approval was required for the conduct of this study.

Outcomes were analyzed according to the intention-to-treat principle at the longest available follow-up. Relative risks (RRs) and 95% CIs were derived from event counts and 2-by-2 tables. RRs were pooled applying inverse variance weighting, with random-effects models fitted using restricted maximum likelihood estimation. Fixed-effects models are also presented. Statistical heterogeneity was explored using the I^2^ statistic and Cochrane’s Q test. *P* values <0.05 (2-tailed) were considered significant.

Seven trials comprising 564 patients (323 randomized to DOAC, 241 randomized to warfarin) were included.^4^, ^5^, ^6^, ^7^, ^8^, ^9^, ^10^ All trials were performed in the setting of acute myocardial infarction (MI), none in nonischemic cardiomyopathy. Rivaroxaban was the most commonly chosen DOAC (k = 4, n = 447) with the remainder investigating apixaban (k = 3, n = 117). All studies were conducted as open-label trials; one trial performed blinded core-lab assessment of thrombus resolution.^9^ Two-dimensional transthoracic echocardiography was universally used for thrombus assessment. The median duration of follow-up was 3 months (IQR: 3-6 months). In patients randomized to warfarin, the median time in therapeutic range was 73% (IQR: 63%-82%). Risk of bias was moderate for most trials (83%), predominantly related to a lack of blinding or core lab-based adjudication, and presentation of results in a per-protocol manner.

Primary findings are summarized in the Figure 1. DOACs were associated with a similar rate of thrombus resolution compared to warfarin, both in random-effects (RR: 1.01; 95% CI: 0.96-1.06; *P* = 0.73; I^2^ = 0%) and fixed-effects models (RR: 1.01; 95% CI: 0.96-1.05; *P* = 0.83). No difference in stroke risk was seen between DOACs and warfarin on random-effects (RR: 0.60; 95% CI: 0.07-5.03; *P* = 0.64; I^2^ = 46%) and fixed-effects analyses (RR: 0.70; 95% CI: 0.17-3.43; *P* = 0.73). In terms of safety, no difference in all-cause mortality was observed on random-effects and fixed-effects analyses (RR: 0.92; 95% CI: 0.37-2.32; *P* = 0.86; I^2^ = 0%). Lastly, major bleeding (RR: 0.49; 95% CI: 0.17-1.45; *P* = 0.20; I^2^ = 0%) and clinically significant nonmajor bleeding (RR: 0.89; 95% CI: 0.20-3.96; *P* = 0.88; I^2^ = 0%) were comparable on random-effects and fixed-effects analysis.

**Figure 1:**
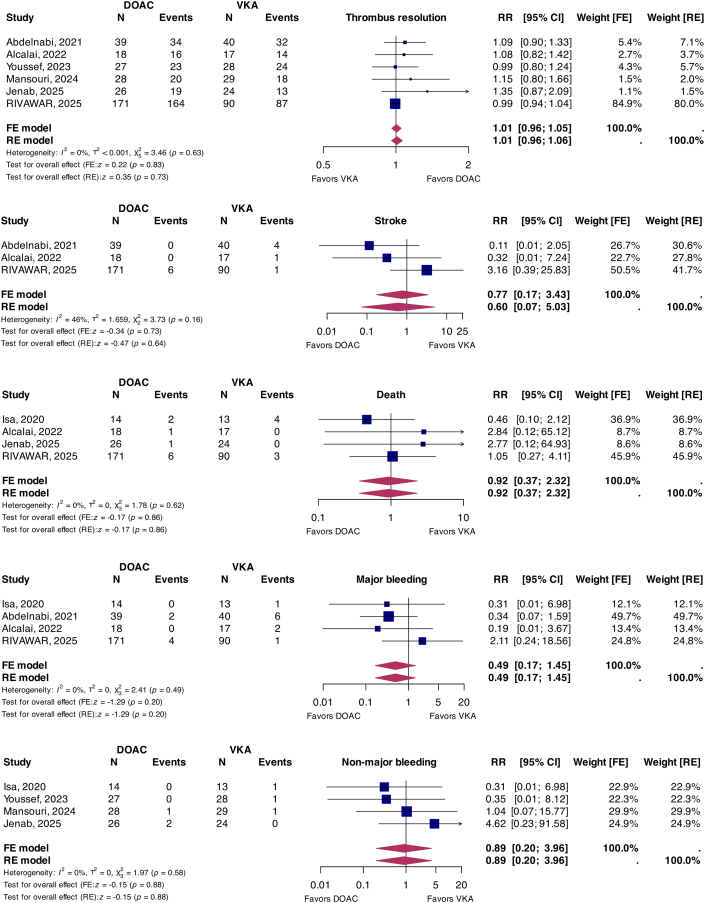
Comparison Between DOACs and VKAs in LV Thrombus Treatment DOACs = direct oral anticoagulants; FE = fixed-effects; LV = left ventricular; RE = random-effects; RR = relative risk; VKAs = vitamin K antagonists.

This meta-analysis synthesizes the totality of randomized evidence on anticoagulation strategies in LV thrombus management. The off-label application of DOACs has gained momentum due to extrapolation of evidence from alternative clinical settings and due to favorable findings from nonrandomized studies.^3^ However, DOACs generated inferior results in patients with mechanical heart valves and LV assist devices, necessitating caution for their expanded use when dedicated randomized evidence is scarce. In the present meta-analysis, DOACs and warfarin demonstrated comparable efficacy in terms of thrombus resolution, while no significant differences were observed for all other endpoints. Results remained consistent across statistical models, and between-study statistical heterogeneity was negligible across outcomes, except for stroke. Since constituent trials were not powered for the assessment of stroke, mortality and bleeding, events were few and CIs were wide, suggesting that—individually and collectively—existing trials may remain underpowered to draw definitive conclusions for these hard endpoints.

This meta-analysis is limited by underlying trial quality, principally featuring open-label designs, small sample sizes, brief follow-up durations, and lacking independent event adjudication. Thrombus assessment was performed using standard transthoracic echocardiography, which has a lower sensitivity for thrombus detection compared to magnetic resonance imaging or contrast-enhanced echocardiography. The limited number of available trials precluded formalized testing for small-study effects using funnel plots or linear regression. Finally, trials were primarily conducted in Asian and African centers and exclusively evaluated ischemic pathologies, thereby impeding generalization of results to European and American cohorts, or patients with nonischemic dilated cardiomyopathy.

In this meta-analysis of RCTs, DOACs and warfarin displayed comparable efficacy in terms of LV thrombus resolution following MI. Larger randomized trials adequately powered for hard clinical endpoints such as stroke and mortality are still required before equivalence or superiority can conclusively be established between these anticoagulation regimes.

## Funding support and author disclosures

Dr Ahmad is a consultant for Cardiovascular Systems Inc and Shockwave; and has served on the Medical Advisory Board of Boston Scientific. Dr Howard reports a relationship with Mycardium AI Limited that includes equity or stocks. Dr Lehmann has received speaker’s honoraria from MSD, Novartis, Daiichi Sankyo, and AstraZeneca and personal fees from Servier Pharmaceuticals and AstraZeneca outside the submitted work. Dr Frey has received speaker fees from 10.13039/100004325AstraZeneca, 10.13039/100015739Bayer Vital, 10.13039/100001003Boehringer Ingelheim, 10.13039/100004330GlaxoSmithKline, 10.13039/100004336Novartis, 10.13039/100004319Pfizer, and Daiichi Sankyo Deutschland none of which related to the content of this manuscript. All other authors have reported that they have no relationships relevant to the contents of this paper to disclose. Dr Reddy is funded by the 10.13039/501100024811Nuffield Department of Population Health, University of Oxford. Dr Howard is funded by the 10.13039/501100000274British Heart Foundation (FS/ICRF/22/26039).

## References

[1] Camaj A., Fuster V., Giustino G. (2022). Left ventricular thrombus following acute myocardial infarction: JACC state-of-the-art review. J Am Coll Cardiol.

[2] Rao S.V., O’Donoghue M.L., Ruel M. (2025). 2025 ACC/AHA/ACEP/NAEMSP/SCAI guideline for the management of patients with acute coronary syndromes: a report of the American college of cardiology/American heart association joint committee on clinical practice guidelines. Circulation.

[3] Haller P.M., Kazem N., Agewall S. (2024). Oral anticoagulation in patients with left ventricular thrombus: a systematic review and meta-analysis. Eur Heart J Cardiovasc Pharmacother.

[4] Shah J.A., Hussain J., Ahmed B. (2025). Rivaroxaban vs warfarin in acute left ventricular thrombus following myocardial infarction. JACC Adv.

[5] Youssef A.A., Alrefae M.A., Khalil H.H. (2023). Apixaban in patients with post-myocardial infarction left ventricular thrombus: a randomized clinical trial. CJC Open.

[6] Isa W.Y.H.W., Hwong N., Mohamed Yusof A.K. (2020). Apixaban versus warfarin in patients with left ventricular thrombus: a pilot prospective randomized outcome blinded study investigating size reduction or resolution of left ventricular thrombus. J Clin Prev Cardiol.

[7] Alcalai R., Butnaru A., Moravsky G. (2022). Apixaban vs. warfarin in patients with left ventricular thrombus: a prospective multicentre randomized clinical trial. Eur Heart J Cardiovasc Pharmacother.

[8] Abdelnabi M., Saleh Y., Fareed A. (2021). Comparative study of oral anticoagulation in left ventricular thrombi (No-LVT trial). J Am Coll Cardiol.

[9] Jenab Y., Sadeghipour P., Mohseni-Badalabadi R. (2025). Direct oral anticoagulants or warfarin in patients with left ventricular thrombus after ST-elevation myocardial infarction: a pilot trial and a prespecified meta-analysis of randomised trials. EuroIntervention.

[10] Mansouri P., Jazi Z.A., Mansouri M.H. (2024). Evaluation of the efficacy and safety of rivaroxaban compared to warfarin in patients with left ventricular apical thrombus: a randomized clinical trial. Thromb J.

